# Metagenomics of Bacterial Diversity in Villa Luz Caves with Sulfur Water Springs

**DOI:** 10.3390/genes9010055

**Published:** 2018-01-22

**Authors:** Giuseppe D’Auria, Alejandro Artacho, Rafael A. Rojas, José S. Bautista, Roberto Méndez, María T. Gamboa, Jesús R. Gamboa, Rodolfo Gómez-Cruz

**Affiliations:** 1 Sequencing and Bioinformatics Service, Foundation for the Promotion of Health and Biomedical Research of Valencia Region (FISABIO), Valencia 46020, Spain; dauria_giu@gva.es; 2 Center for Advanced Research in Public Health, Foundation for the Promotion of Health and Biomedical Research of Valencia Region (FISABIO), Valencia 46020, Spain; artacho_ale@gva.es; 3 Chemical Engineering Faculty, Exact Sciences and Engineering Campus, Autonomous University of Yucatán (UADY), Mérida, Yucatán 97050, Mexico; rafael.rojas@correo.uady.mx; 4 Biological Sciences Academic Division, Autonomous University Juárez de Tabasco (UJAT), Villahermosa, Centro, Tabasco 99630, Mexico; jsantiagobautistal1@gmail.com (J.S.B.); biol.rmendezperez@gmail.com (R.M.); gambtere@gmail.com (M.T.G.); robertogamboa@usa.net (J.R.G.)

**Keywords:** metagenomics, bTEFAP, *Proteobacteria*, *Acidobacteria*

## Abstract

New biotechnology applications require in-depth preliminary studies of biodiversity. The methods of massive sequencing using metagenomics and bioinformatics tools offer us sufficient and reliable knowledge to understand environmental diversity, to know new microorganisms, and to take advantage of their functional genes. Villa Luz caves, in the southern Mexican state of Tabasco, are fed by at least 26 groundwater inlets, containing 300–500 mg L^−1^ H_2_S and <0.1 mg L^−1^ O_2_. We extracted environmental DNA for metagenomic analysis of collected samples in five selected Villa Luz caves sites, with pH values from 2.5 to 7. Foreign organisms found in this underground ecosystem can oxidize H_2_S to H_2_SO_4_. These include: biovermiculites, a bacterial association that can grow on the rock walls; snottites, that are whitish, viscous biofilms hanging from the rock walls, and sacks or bags of phlegm, which live within the aquatic environment of the springs. Through the emergency food assistance program (TEFAP) pyrosequencing, a total of 20,901 readings of amplification products from hypervariable regions V1 and V3 of 16S rRNA bacterial gene in whole and pure metagenomic DNA samples were generated. Seven bacterial phyla were identified. As a result, *Proteobacteria* was more frequent than *Acidobacteria*. Finally, acidophilic *Proteobacteria* was detected in UJAT5 sample.

## 1. Introduction

The tropical rainforest is one of the world’s main biomes, biologically possessing the greatest wealth of the tropics [1,2,3], but also the most endangered [4]. The Mexican humid tropics (MHT) or warm-humid tropics is in the southeast region of Mexico. It occupies just 11% of the country’s landscape [5], but it has the greatest biological diversity [6]. One of the characteristic epicontinental ecosystems in the MHT are underground aquatic biomes, which are interconnected with springs and the Chichonal volcano by an aquifer network that spans hundreds of kilometers in a region lying North of the Sierra de Chiapas in southeastern Mexico [7]. Thus, Villa Luz (VL) caves are characterized by the presence of elemental sulfur embedded in the walls with 300–500 mg L^−1^ H_2_S and <0.1 mg L^−1^ O_2_ in the air, as well as karst springs and colloidal sulfur [8,9,10,11]. H_2_S concentration in the atmosphere varies and is often quite toxic. Besides the fascinating hydrology and atmosphere, VL caves have a diverse biological community that appears to be largely dependent on the mineral-rich waters [8]. This is a very special environment, as it has a mix of energy resources such as bat guano, plant debris, and most extraordinary of all, autotrophic bacteria colonies.

The influence of the bacterial diversity on the cave’s development is very evident as H_2_S oxidation takes place in the clay, which is replaced by gypsum. Gypsum falls apart and dripping water dissolves it, which makes the replacement-solution phenomenon very evident [12]. Because of its early origin (Cretaceous) and extreme environmental conditions (pH, temperature, Sulphur compound concentrations, and redox conditions), like those found in the Early Earth [7], these caves house a biological library that is not very well explored from the molecular point of view.

Overall, it can represent an opportunity to develop biotechnological applications first, but also to gain insights into the origin and evolution of mechanisms of survival in extreme environments. Biodiversity characterization of native microorganisms is of enormous biological and ecological importance, and it also has a great impact on biotechnological potential [13], because they are the most abundant lifeform on earth [14] and they are also indispensable for the functioning of any ecosystem [8,15,16]. 

Moreover, there are few biological and native microbial genetics richness studies of Earth’s ecosystems in relation to the vast microbial diversity existing, which could represent up to 99% of non-cultivable microorganisms of all individuals present in an environment [17], with Archaea and bacteria being among them [18,19,20]. Bacteria thrive in a wide variety of habitats, they are an important part of global ecosystems and they are considered to represent up to 20% of the Earth’s total biomass [21,22,23]. Taking into account the huge biotechnological potential concealed in bacteria, so far very poorly characterized, to analyze their genomic contents through metagenomic tools does open the possibility of identifying new taxa or novel genes. In addition to underlining their role in the ecosystem, these have the potential to be applied in the food, pharmaceutical, organic chemical industries [22] or other uses [24,25]. 

Metagenomics, as a next-generation sequencing (NGS) field, offers a modern way to determine community structure, species diversity, metabolic capacity, and functional diversity studies [3,26,27]. The NGS technologies, including 454 and Illumina sequencers, use oligonucleotides to amplify the *rrs* gene encoding for the 16S rRNA subunit and are targeted to hypervariable regions. Although no single hypervariable region can distinguish among all the bacteria, V2 (nucleotides 137–242), V3 (nucleotides 433–497), and V6 (nucleotides 986–1,043) hypervariable regions contain the maximum heterogeneity and provide the maximum discriminating power for analyzing bacterial groups. Furthermore, the fact that hypervariable regions are flanked by conserved regions and known sequences allows to design specific oligonucleotides. These allow to amplify the sites or fragments by polymerase chain reaction (PCR), and to sequence them by NGS in order to identify and quantificate the microbial diversity [18,28].

In this work, we sequenced the 16S rRNA genes of environmental DNA acquired from the native microbiota of the VL caves in Tacotalpa, Tabasco, Mexico. The sequence analysis of metagenomic data, generated by massive bacterial tag-encoded FLX amplicon pyrosequencing (bTEFAP), is meant to identify the bacterial diversity, at a family level, and can be of help for future biotechnological implications.

## 2. Materials and Methods 

**Figure 1:**
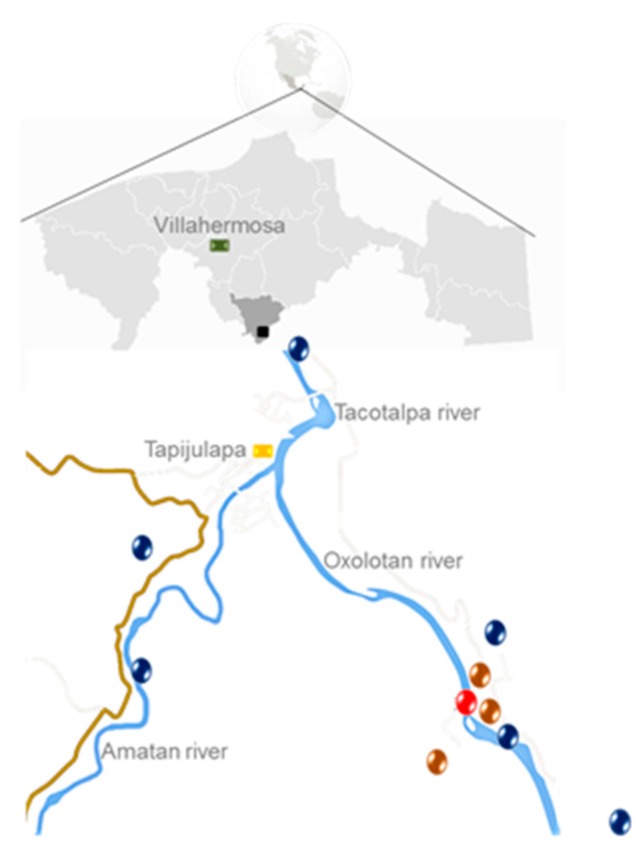
Geographical location of the Villa Luz (VL) caves, Tacotalpa, Tabasco, Mexico. The blue spots indicate no sulfidic surface bodies, brown spots indicate sulfidic surface sites, and the red spot shows the Sulphur cave (CA) sulphidic. Adapted from Plath, M.; Tobler, M. CRC Press Taylor & Francis Group. 2010, Chapter 8, p. 285. Copyright 2018 CRC Press. [9].

The VL caves, also known as Sardines or Sulfur caves, are formed by sulfur-rich waters of hypogenic origin. These caves are located in the municipality of Tacotalpa, Tabasco, Mexico (3.5 km south of Tapijulapa), at 17°28′0″ N, 92°47′0″ W coordinates, and are within Kolem Jaa Park located at an altitude of 100 meters above sea level. These caves are approximately 2.4 km from Almandro River, located at the Chiapas high edges. They have a total surveyed length of approximately 1.9 km and the total relief of the explored caves is just 25 km, see Figure 1 [8,9,12]. VL caves water flows about 80 m above sea level and 40 m, approximately, on the hydrological base level, which is represented by Oxolotán and Amatán rivers [8]. This system is composed of two caves: Cueva del Azufre (Sulphur cave), that is fed by about 26 sulfidic springs, and Cueva Luna Azufre (Sulfur Moon cave), with non-sulfidic springs [8,12]. 

**Figure 2:**
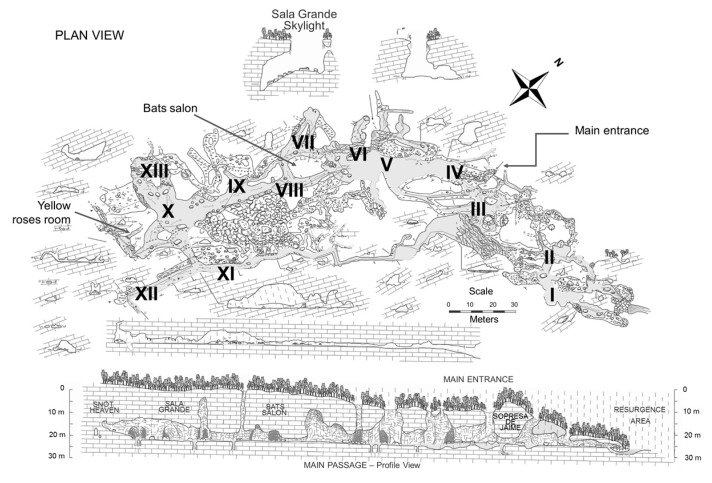
Simplified map of the VL caves, indicating the location of springs (dark spots), skylights, broken limestone columns, main entrance, and areas with elemental sulfur. It also shows the I–XIII cameras and their location. Adapted from Hose, L.D., Pisarowicz, J.A., Journal of Cave and Karst Studies. 1999, vol. 61, 1, pp. 13–21. Copyright 2017, National Speleological Society, Inc. (NSS). [10].

These caves are divided into 13 different chambers (I–XIII) [10,29], see Figure 2. They were formed by the folding of a micritic block of limestone in the Cretaceous period and are limited to the South by a normal fault, which probably controls the location of its main entrance. Front chambers receive a certain amount of light through the roof skylights, while the more inner chambers are completely dark. Several anastomosing streams that flow through the cave are fed by springs that emerge from the limestone floor, most of which contain H_2_S and possibly gas bubbles with CO_2_. Based on the chemistry and physical nature, the springs have been classified into two groups. Group A members are characterized by containing between 300 and 500 mg L^−1^ H_2_S, and less than 0.1 mg L^−1^ O_2_. This water is slightly oversaturated with calcite and oversaturated with gypsum and dolomite. It is recognizable in the caves by the elemental sulfur contained in the walls above the high-water mark, the white bacterial filaments on the wet rock surfaces, and the pyrite deposits in the water-covered sediments or rocks. Group B springs have <0.1 mg L^−1^ H_2_S and ≤4.3 mg L^−1^ O_2_. They are characterized by travertine and iron oxides (red-yellow color) precipitation, calcite and dolomite oversaturation, and gypsum sub-saturation. Both type (AB-type) springs have elements of the first two spring groups and their composition is the most abundant in these caves. Based on total dissolved solids and chemistry in general, it has been proposed that the A and B groups have a similar origin and composition, suggesting that H_2_S oxidation takes place first in group B. The causes or controls on water oxidation are still unknown [7,8,9,10].

### 2.1. Environmental Sampling and DNA Extraction for Metagenomic Analysis

**Figure 3:**
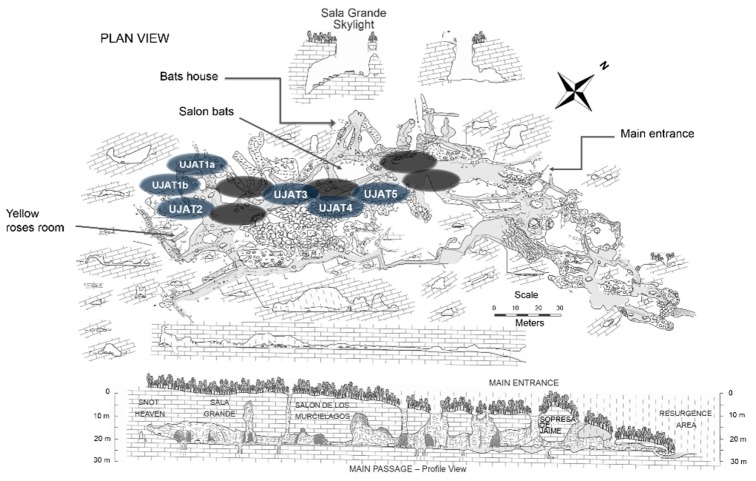
Pre-selected and selected sites for the environmental samples collection in the VL caves. Elliptical shapes marked and labeled from UJAT1 to UJAT5. Adapted from Hose, L.D., Pisarowicz, J.A., Journal of Cave and Karst Studies. 1999, vol. 61, 1, pp. 13–21. Copyright 2017, National Speleological Society, Inc. (NSS) [10].

Snottite, biovermiculite, and sediment samples were collected from 10 pre-selected sites (Figure 3), considering their physicochemical characteristics (temperature, pH, and H_2_S smell). These samples were transported to laboratory in a thermal cooler, in which they were pre-frozen with liquid nitrogen and stored at −40 °C until subsequent DNA extraction. Metagenomic DNA from 0.5g of environmental samples was extracted using the FastDNA^®^ SPIN Kit for Soil (MP Biomedicals, Santa Ana, CA, USA), following the manufacturer’s recommendations. Snottite samples were frozen with liquid nitrogen and then were ground to achieve better disintegration of the embedded cells within the mucus-like layers and to facilitate mechanical and chemical lysis. Sediment samples were centrifuged at 10,000× g for 20 min with a centrifuge 5810R (Eppendorf, Hamburg, Germany) to completely extract excess water and precipitate bacterial cells. As for the biovermiculites samples, the DNA was directly extracted. The DNA obtained was used to amplify 1400 base pairs (bp) of bacterial 16S ribosomal DNA (rDNA) gene using the oligonucleotides: NVZF 5′-GCG GAT CCG CGG CCG CTG CAG AGT TTG ATC CTG GCT CAG-3′ and NVZR 5′-GGC TCG AGC GGC CGC CCG GGT TAC CTT GTT ACG ACT T-3′ [30]. The PCR reaction mix consisted of 50 ng DNA template, 1x PCR buffer (Qiagen, Hilden, Germany), 0.025M MgCl_2_ (Merck, Darmstadt, Germany), 200 μM each deoxynucleotide (dNTP) (Promega, Madison, Wisconsin, USA), 0.6 μM each oligonucleotide, and 0.5U HotStarTaq DNA Polymerase (Promega). PCR reaction conditions were: an initial denaturation step of 94 °C for 10 min followed by 30 cycles at 94 °C for 1 min, and 58 °C for 1 min, 72 °C for 1 min, and a final extension at 72 °C for 10 min, with a T-gradient Thermo cycler, the CFX96 Touch^TM^ Real-Time PCR (Bio-Rad, Singapore, Singapore). The PCR products were evaluated by submerged electrophoresis in 1.2% agarose gel stained with ethidium bromide and quantified using a NanoDrop 2000 Spectrophotometer (Thermo Scientific, Waltham, MA, USA). Finally, they were diluted to 100 ng μL^−1^ by bTEFAP analysis, for which the Research and Testing Laboratory services (Lubbock, TX, USA), were required.

### 2.2. Pyrosequencing and Sequence Analysis

Clone library and pyrosequencing preparation services were requested from the Research and Testing Laboratory. To perform PCR we used specific universal primers 28F (5′-GAGTTTGATCNTGGCTCAG-3′) and 519R (5′-GTNTTACNGCGGCKGCTG-3′) of the 16S rRNA gene V1 and V3 variable regions. A systematic check was performed to remove low-quality reads in accordance with Brown et al.’s (2009) strategies [31]. These involve eliminating: (i) Sequences that do not perfectly match the 3bp key code and primer sequence at the start of read, (ii) Sequences that do not perfectly match at least the first 10bp of the distal primer, (iii) Sequence reads that contain any undetermined nucleotide (N), and (iv) Sequence reads <50 bp after removing both primers [32]. The data was obtained using an ad hoc channel written in Perl. All statistics were obtained with RStatistics software, making use of several open-source libraries such as GData [33] and Vegan [34]. The group sequences were calculated to have 0.97% of similarity and 80% of overlap by using the Cluster Database at High Identity with Tolerance (CD-HIT) software [35]. Taxonomic affiliations were assigned using the Ribosomal Database Project (RDP) classifier [36] and all data were tabulated. Readings with RDP score value <0.8 were assigned below the taxonomic rank/range and left in the last rank as unidentified.

## 3. Results

### 3.1. Samples and Chemical Properties

Samples from five different sites in the VL caves were selected (UJAT1, UJAT2, UJAT3, UJAT4, and UJAT5). As extreme values, we measured from 27 °C to 30 °C, while the pH was found to be from 2.5 to 7. As seen in Table 1, UJAT1 and UJAT2 samples contained high chemical concentrations, UJAT4 and UJAT5 samples contained intermediate chemical concentrations, and the UJAT3 sample was obtained from a microenvironment with low chemical concentrations, mainly CO_2_, CH_4_, and H_2_S. The UJAT5 sample represents an acidophilus microniche (pH 2.5).

### 3.2. Bacterial Diversity Distribution

The pyrosequencing studies with V1–V3 hypervariable regions of bacterial 16S rRNA gene by PCR amplified from five selected sites provided 20,901 readings with an average size of 434.2 bp (standard deviation (SD) average: 55.3). Using the Chao 1 estimator [33,34,38], the taxonomic analysis of sequences revealed the presence of 27 and 81 families in the UJAT2 and UJAT1b samples, respectively. According to the Shannon Index, the diversity had all values >1, with a maximum of 3.02 for UJAT1b sample. UJAT3 sample reported index of 0.73. See Table 2. 

**Figure 4:**
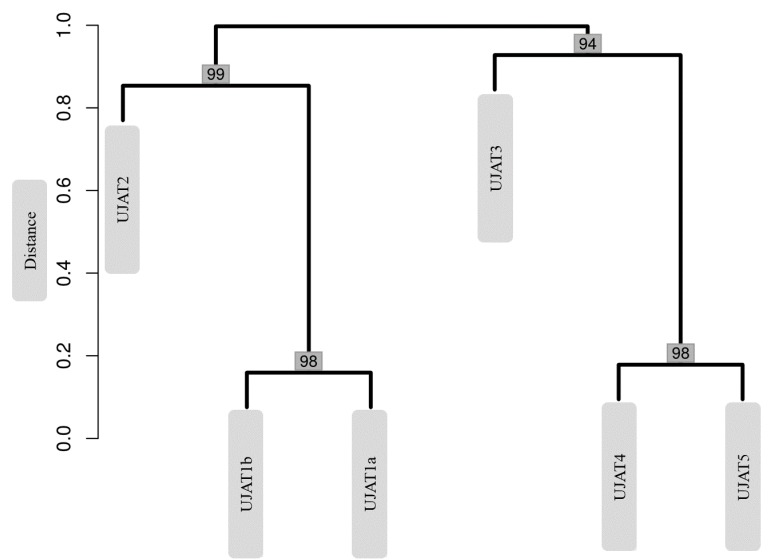
Dendogram resulting from the complete cluster analysis based on families’ distribution. Numbers at the branching points represents bootstrap values (percentage over 1000 replicates).

These high Shannon index values indicate a diversity-balanced distribution. Only in the UJAT3 sample we found a diversity decrease, corresponding to increased dominance of *Enterococcaceae* and *Anaerolineaceae* members (Figure 4). Samples are grouped by sampling location, as displayed in Figure 4, which shows that the UJAT1a and UJAT1b samples closely resemble each other, together with the UJAT2 sample. Then, the UJAT4 and UJAT5 samples are of another cluster separate from the UJAT3 sample and of the first mentioned samples. 

**Figure 5:**
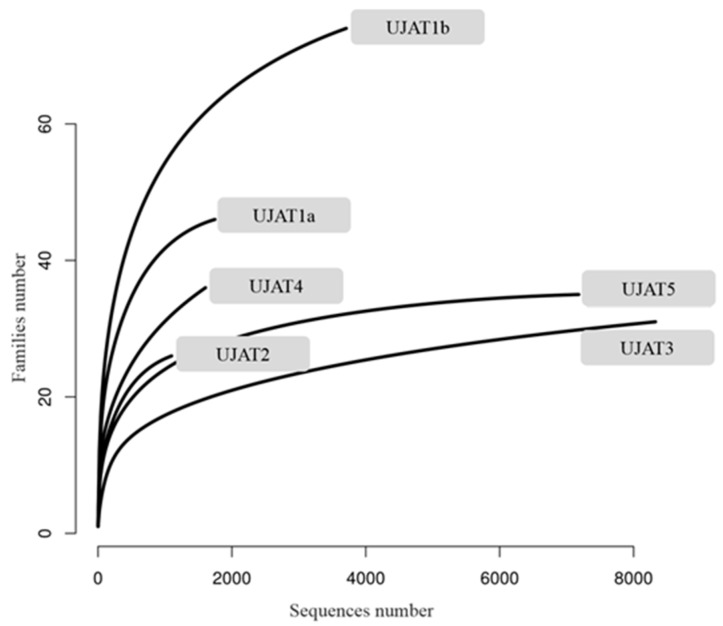
Rarefaction curves of 16S rRNA amplicons among samples. X axis defines number of reads; Y axis defines, percentage-wise, the distribution and abundance of families per sample.

Rarefaction curves obtained at family level, indicate that all samples except UJAT4, where the estimated Chao1 indicates the presence of up to 81.1 families (standard error (SE): 6.13), tend to reach a plateau, which indicates that the sequencing depth was sufficient to carry out a thorough description of each sample (Figure 5).

**Figure 6:**
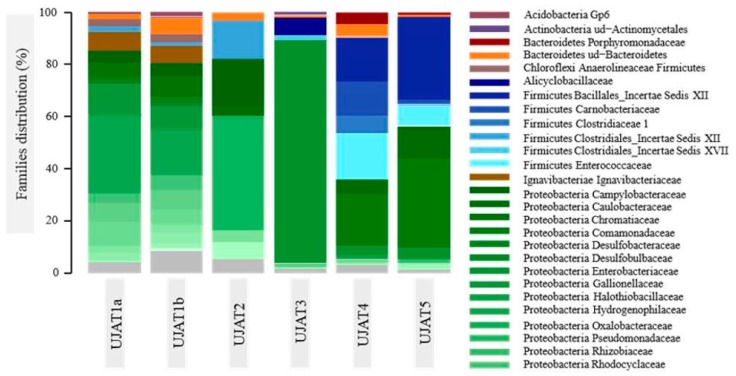
Relative frequency families from selected sites in VL caves.

From Figure 6 we see that UJAT1a and UJAT1b samples closely resemble each other, and a bit the UJAT2 sample, while the UJTA3, UJAT4 and UJAT5 are more different. Taxonomic analysis revealed the presence of seven bacterial phyla: *Acidobacteria*, *Actinobacteria*, *Bacteroidetes*, *Chloroflexi*, *Firmicutes*, *Ignavibacteria* and *Proteobacteria*. *Proteobacteria* was the most abundant phylum in collected UJAT1a, UJAT1b, UJAT2, and UJAT3 samples of selected sites (82.85–89.38%, Figure 6). However, in UJAT4 and UJAT5 samples, the most abundant is *Firmicutes* (58.66% and 42.56%, respectively), while the *Proteobacteria* represented 35.49% and 56.49%, respectively. 

#### 3.2.1. *Proteobacteria*

The UJAT1a, UJAT1b, UJAT2, UJAT3, UJAT4, and UJAT5 samples contained 78%, 66%, 69%, 88%, 34%, and 54%, respectively. Among them, the most abundant classes were *Betaproteobacteria* (UJAT1a, UJAT1b, UJAT4, and UJAT5 samples) representing up to 50% of all readings in the UJAT1a sample. In *Betaproteobacteria*, the most recurrent genera were *Thiobacillus* (28% UJAT1a and 14% UJAT1b samples) and *Sideroxydans* (13% UJAT1a and 8% UJAT1b samples), as well as *Rhodocyclaceae* members (5% UJAT1a and 7% UJAT1b samples) and *Delftia* (18% UJAT4, 32% UJAT5 samples). *Gammaproteobacteria* was the most representative class in the UJAT3 sample, where *Serratia* represented almost all the readings (84%). In the UJAT2 sample, *Gammaproteobacteria* were also very abundant, reaching up to 37% of the readings, and between the dominant taxa were *Pseudomonas* (36%), *Moraxellaceae* (0.2%), and *Azotobacter* (1%). The UJAT1a, UJAT1b, and UJAT2 samples were also characterized by 15%, 11%, and 16% of *Epsilonproteobacteria*, and especially *Campylobacterales* members of *Arcobacter* and *Sulfurovum* genera (12%, 5%, and 1%) in the UJAT1a sample; *Dehalospirillum*, *Arcobacter*, and *Helicobactreaceae* (5%, 4%, and 1%) in the UJAT1b sample; and *Sulforospirillum* and *Arcobacter* (17% and 5%) in the UJAT2 sample. The *Epsilonproteobacteria* were practically absent in the other selected samples. With regards to *Alphaproteobacteria*, they were mainly found in the UJAT2, UJAT4, and UJAT5 samples (9%, 6%, and 15%, respectively). The most representative orders were *Rhizobiales* and *Caulobacteriales*. In the UJAT2 sample, the *Rhizobiales* (6%) were represented by *Rhizobium* (4%) and *Hoeflea* (2%); in *Caulobacteriales* (3%), by *Caulobacter* (2%) and *Brevundimonas* (1%). The majority of *Alphaproteobacteria* in the UJAT4 sample were *Caulobacteriales* (5%), composed entirely of *Brevundimonas* and *Rhizobiales* (0.7%) with *Rhizobium*. In the other samples, the *Alphaproteobacteria* were almost absent.

#### 3.2.2. *Firmicutes*

These were represented by values lower than 20% in all samples, except the UJAT4 and UJAT5 samples. The UJAT1a and UJAT1b samples (2% and 3%, respectively) were represented almost entirely by Clostridiales orders (2% and 2%), and mostly by *Fusibacter* genus and other minor genera. Likewise, *Lactobacillales* and *Bacillales* order (<1%) were found. The UJAT2 sample was composed by 17% of this phylum with 15% *Clostridia* class, including 12% represented by *Fusibacter*; other orders such as *Selenomonadales* with *Succiniclasticum* (2%) and Bacilli class with *Planomicrobium* genus (0.5%) were found present. In the UJAT3 sample, this phylum was represented by 9%, with 8% of Bacilli class almost entirely represented by *Alicyclobacillus* genus, and 2% of *Clostridia* class fully composed by *Sulfobacillus* genus. *Firmicutes* in the UJAT4 and UJAT5 samples were present, 53% and 41%, respectively. In the UJAT4 sample, Bacilli and *Clostridia* members (45% and 7%, respectively) were found. Likewise, among Bacilli class, *Exiguobacterium* (16% and 31%), *Enterococcus* (17% and 8%), and *Trichococcus* (12% and 4%) genera were found. Finally, in *Clostridia* class, we identified *Proteinoclasticum* (2% and 0.2%) and *Clostridioides* genus *in stricto sensu* (4%; absent in the UJAT-5 sample).

#### 3.2.3. Others

Other organisms found in the UJAT1a and UJAT1b samples were *Ignavibacterium* (8% and 7%, respectively), *Chloroflexi* (4% and 4%, respectively), *Acidobacteria* (2% and 5%, respectively), and *Bacteroidetes* (3% and 6%, respectively). In the UJAT2 sample, *Actinobacteria* (2%), Lentisphaerae (2%), and Bacteroidetes (2%) were also found. In the UJAT4 and UJAT5 samples, *Bacteroidetes* by 10% and 2%, respectively, were found. In these samples, the represented genera of the phylum *Bacteroidetes* were *Paludibacter* (10% and 2%, respectively), and other minor genera represented (<1%) were *Parabacteroides*, *Thermophagus*, *Alkalitalea*, *Alkaflexus*, *Moheibacter*, *Wandonia*, and *Meniscus*, among others.

## 4. Discussion

The number of metagenomic studies has increased in recent years [39]. Metagenomics has been used to evaluate and exploit biodiversity in many habitats, including extremophiles environments [40,41,42,43,44,45]. In this study, we determined the prokaryotic diversity of sulphydric hot springs in the VL caves of Tacotalpa, Tabasco, Mexico, with severe limitations or total absence of light. We found different bacterial communities that were dominated by *Proteobacteria*, *Firmicutes*, *Chloroflexi*, *Chlorobi*, *Bacteroidetes*, and *Actinobacteria*. We also found the phylum *Acidobacteria*, although with very little dominance. A dominance of *Proteobacteria* was observed in this study and is in accordance with other cave studies [46,47]. This suggests that the presence of this community is a consequence of the increase in organic matter entering this cave [47]. Although the interaction of these bacteria might develop metabolic capability against possible contamination by infiltration of human or animal organic matter [48], the bat guano could be the main source of organic matter responsible for making *Proteobacteria* the dominant phylum in this cave. Dominance and pH found in the UJAT5 sample microbiota (Figure 4; Table 1) suggest that this might correspond to acidophilic *Proteobacteria*, which in the case of iron oxidizers has been the focus of a large amount of research due to its significance in environmental biotechnology [49]. 

Gram-positive *Firmicutes*, *Bacteroidetes*, and *Chloroflexi* bacterial phyla are described as follows: *Firmicutes* are present in all aquatic environments, *Bacteroidetes* are green but not sulfurous, and *Chloroflexi* have low abundance in oligotrophic waters [50] . Wemheuer et al. (2013) were focused on the evaluation and exploitation of the prokaryotic diversity in two microbial communities obtained from different hot springs in Kamchatka; using the metagenomic approach, they found that the most abundant groups in the samples belonged to *Proteobacteria*, *Thermotogae*, and *Thaumarchaeota*, but they did not find *Acidobacteria*. This phylum is widely distributed and is abundant in soils, it is not restricted to acidic environments and is made up of oligotrophic organisms negatively correlated with soil organic matter [51]; however, their ecological and metabolic functions are not accurately known, because we do not have pure cultures neither do we have complete genomes sequences [30,52,53,54,55]. *Acidobacteria* phylum is identified by a diverse collection of 16S rRNA gene sequences (>1500 in the Data Base Project ribosome [24] obtained from different environments, including soils and sediments [56,57], soil crusts of sand dunes [58], sewage [59,60], sewage distribution systems [61], mire or quagmire [62], acid mine drainage [63], intertidal hot springs [37], submarine hydrothermal vents shallow [64], surfaces Paleolithic rock paintings and catacombs [65,66,67,68], and interactions of species of this phylum with plants [43]. In situ hybridization with specific probes for *Acidobacteria* has also confirmed the presence of this phylum in many environments and revealed multiple cellular morphotypes, including cocci, short rods, and thin filaments [69]. Numerous 16S rRNA gene sequences from this phylum have also been identified in different active and ancient cave systems worldwide [70]. However, our knowledge of acidobacterial diversity is still rather incomplete [71], and even more considering only from caves [70]. 

Finally, our study revealed that all the bacteria identified herein are characteristic of caves and, in the *Acidobacteria*, *Proteobacteria*, and *Actinobacteria* cases, they are predominant bacterial communities on volcanic terrain [72], as is the case with this cave, which is very close to the Chichonal volcano. Currently, it is becoming widely understood that speleogenesis is induced by sulfuric acid, where sulfuric acid causes the dissolution of limestone and results in the precipitation of gypsum, a fact that has been implicated in the formation of numerous caves [8,9,12]. However, there are very few studies on the role of bacteria in the speleogenesis induced by sulfuric acid and its metabolism in this environment type.

## 5. Conclusions

To sum up, 20,901 reads of bacterial 16S rRNA gene sequences spanning V1–V3 hypervariable regions corresponded to seven phyla: *Proteobacteria*, *Firmicutes*, *Chloroflexi*, *Chlorobi*, *Bacteroidetes*, *Actinobacteria* and *Acidobacteria*. *Proteobacteria* phylum dominance could be due to the increased presence of organic matter, not only of bat guano but also that caused by man and animals, directly or through infiltrations. For the UJAT5 sample, we generated 6691 reads, which, due to the physicochemical characteristics (Table 1) and relative frequency obtained (Figure 4), could confirm the presence of acidophilic *Proteobacteria* in VL caves. All the bacterial communities identified are characteristic of caves, while *Acidobacteria*, *Proteobacteria*, and *Actinobacteria* are typical of volcanic surface terrain.

## Figures and Tables

**Table 1:** Physicochemical measurements of the sampling sites selected of VL caves.

Sample Type	Selected Sampling Site in Vila Luz cave	Physicochemical Measurements									
		pH	T (°C)	ppmv in the Atmosphere		Concentration (mg L^−1^)					
				CO_2_	CH_4_	SO_4_^2−^	Ca^2+^	Mg^2+^	Na^+^	HCO_3_^−^	H_2_S
Sediment-water	UJAT1a	7.0	30	527	2.09	>960	>393	>85.8	>479	>1330	>500
	UJAT1b		28.7			960	~393	85.8	479	1330	500
	UJAT2	6.8	28.6	847	2.63–2.70			75.7–83.8	412–465		
	UJAT3	6.9	27	~396	1.87	<960	~387				
Biovermiculites	UJAT4	7.0		~467	1.97					1310	300
Snottites	UJAT5	2.5						85.8	479		400
Reference		This work		[20]		[27]					[37], this work

ppmv: parts per million volume.

**Table 2:** Estimation of biodiversity and richness of selected sampling sites of VL caves.

Sample	Reads	Length (SD)	N Families	Shannon	Chao1 (SE)
UJAT1a	1686	471.58 (54.77)	47	2.67	47.00 (1.77)
UJAT1b	3286	428.85 (56.72)	81	3.02	81.09 (6.13)
UJAT2	995	424.71 (53.74)	27	2.00	27.00 (2.16)
UJAT3	6842	380.98 (46.38)	36	0.73	36.06 (7.48)
UJAT4	1401	479.87 (62.95)	47	2.41	47.00 (12.47)
UJAT5	6691	419.73 (57.74)	35	1.88	35.12 (0.68)

SD: Standard deviation; N: Number; SE: Standard error.
